# Proof of principle study: synchrotron X-ray fluorescence microscopy for identification of previously radioactive microparticles and elemental mapping of FFPE tissues

**DOI:** 10.1038/s41598-023-34890-6

**Published:** 2023-05-13

**Authors:** Letonia Copeland-Hardin, Tatjana Paunesku, Jeffrey S. Murley, Jasson Crentsil, Olga Antipova, LuXi Li, Evan Maxey, Qiaoling Jin, David Hooper, Barry Lai, Si Chen, Gayle E. Woloschak

**Affiliations:** 1 Department of Radiation Oncology and Department of Radiology, Feinberg School of Medicine, Northwestern University, 300 E. Superior St., Tarry 4-713, Chicago, IL 60611 USA; 2 X-Ray Science Division, Advanced Photon Source, Argonne National Laboratory, Lemont, IL USA; 3 Nuclear Nonproliferation Division, Oak Ridge National Laboratory, Oak Ridge, TN USA

**Keywords:** Biological techniques, Medical research, Materials science

## Abstract

Biobanks containing formalin-fixed, paraffin-embedded (FFPE) tissues from animals and human atomic-bomb survivors exposed to radioactive particulates remain a vital resource for understanding the molecular effects of radiation exposure. These samples are often decades old and prepared using harsh fixation processes which limit sample imaging options. Optical imaging of hematoxylin and eosin (H&E) stained tissues may be the only feasible processing option, however, H&E images provide no information about radioactive microparticles or radioactive history. Synchrotron X-ray fluorescence microscopy (XFM) is a robust, non-destructive, semi-quantitative technique for elemental mapping and identifying candidate chemical element biomarkers in FFPE tissues. Still, XFM has never been used to uncover distribution of formerly radioactive micro-particulates in FFPE canine specimens collected more than 30 years ago. In this work, we demonstrate the first use of low-, medium-, and high-resolution XFM to generate 2D elemental maps of ~ 35-year-old, canine FFPE lung and lymph node specimens stored in the Northwestern University Radiobiology Archive documenting distribution of formerly radioactive micro-particulates. Additionally, we use XFM to identify individual microparticles and detect daughter products of radioactive decay. The results of this proof-of-principle study support the use of XFM to map chemical element composition in historic FFPE specimens and conduct radioactive micro-particulate forensics.

## Introduction

Internal body exposure to nuclear fission products through inhalation or other routes of exposure may occur during mass casualty radiation events. Internal radionuclide contamination was found in tissues from atomic bomb victims^1^. However, studies that delineate the differences between biological effects of external radiation and internal, radionuclide-mediated radiation exposure remain limited. Biobanks housing formalin-fixed, paraffin-embedded (FFPE) tissues from major radiobiology animal studies remain an underutilized resource for investigating the molecular effects of internal exposure^2,3^. The volume of available samples, capacity to repeat studies, ease of storage, and ability to complete retrospective analyses on rare samples make FFPE specimens beneficial for radiobiology research. From the 1950s to the 1990s, the US Department of Energy and its predecessors funded lifespan studies that investigated the effects of radiation exposure on the health of beagle dogs and other animal species^4,5^. The Inhalation Toxicology Research Institute (ITRI) conducted a subset of these experiments which involved exposing dogs to single nuclear fission products including beta-emitting yttrium-90 (^90^Y)^6–9^. These radionuclides were adsorbed to insoluble aluminosilicate particles and delivered to dog lungs via inhalation (Fig. S1). Most of these tissues now reside at Northwestern University as a part of the Northwestern University Radiobiology Archive (NURA)^3,10,11^.

Exposure to ionizing radiation alters the concentrations of trace elements present in serum, intestines, and heart suggesting a role for ionizing radiation in shaping the metallome^12–14^. In recent years, interest in metallomics and elementalomics increased because trace elements have been shown to be essential for many biological functions and imbalance often leads to detrimental biological consequences. For example, enzymes that rely on metal cofactors are integral to mitochondrial metabolism^15^. Additionally, the accumulation of iron and copper in amyloid- β plaques associated with Alzheimer’s disease^16–18^ or depletion of zinc in human or canine prostate tumor tissue^19,20^ suggest that characterizing the metallome may provide insight into disease progression. Therefore, evaluating elemental concentrations in tissues of exposed animals may elucidate the effects of internal radiation exposure in addition to discovering distribution of internal emitters, their daughter products or vehicles used for their delivery.

Synchrotron-based X-ray fluorescence microcopy (XFM) is a highly sensitive, analytical, and semi-quantitative technique used to characterize the spatial distribution and relative concentrations of elements. The non-destructive nature of XFM makes it an ideal technique for sequential imaging of FFPE samples at different XFM instruments as previously demonstrated^19–23^. The Bionanoprobe (BNP), a high-resolution X-ray fluorescence microscope, has a spatial resolution as high as 30 nm which allows for the production of nanoscale elemental maps of cells and subcellular structures^21,22,24–34^. To date, the BNP has not been utilized to characterize elemental maps of archival FFPE tissues.

Here, we completed a proof-of-principle demonstration of the feasibility of XFM instruments with different resolutions, including the BNP, to produce elemental maps of canine FFPE lung and lymph node samples that are approximately 35 years old and contain formerly radioactive micro-particulates. We identified potential aggregates as well as individual aluminosilicate microparticles to which ^90^Y was adsorbed by mapping distribution of silicon (Si), a component of the fused aluminosilicate (FAP) microparticles, and zirconium (Zr), a daughter product of ^90^Y decay. We also completed a comparative analysis of elemental signatures collected with low, medium, and high resolution XFM, using beamlines 8BMB, 2IDE, 2IDD and the BNP located at the Advanced Photon Source (APS) at Argonne National Laboratory (ANL). Interestingly, in all low- and medium-resolution scans, we found close associations between iron signal, a potential marker of macrophages, and silicon signal; the signals becoming spatially distinct only at a high resolution in BNP generated maps where subcellular structures are the most clearly delineated. Future studies will investigate if macrophages transport microparticles to other organs and utilize synchrotron-based XFM to analyze how internal radiation exposure influences the metallome.

## Results

**Figure 1:**
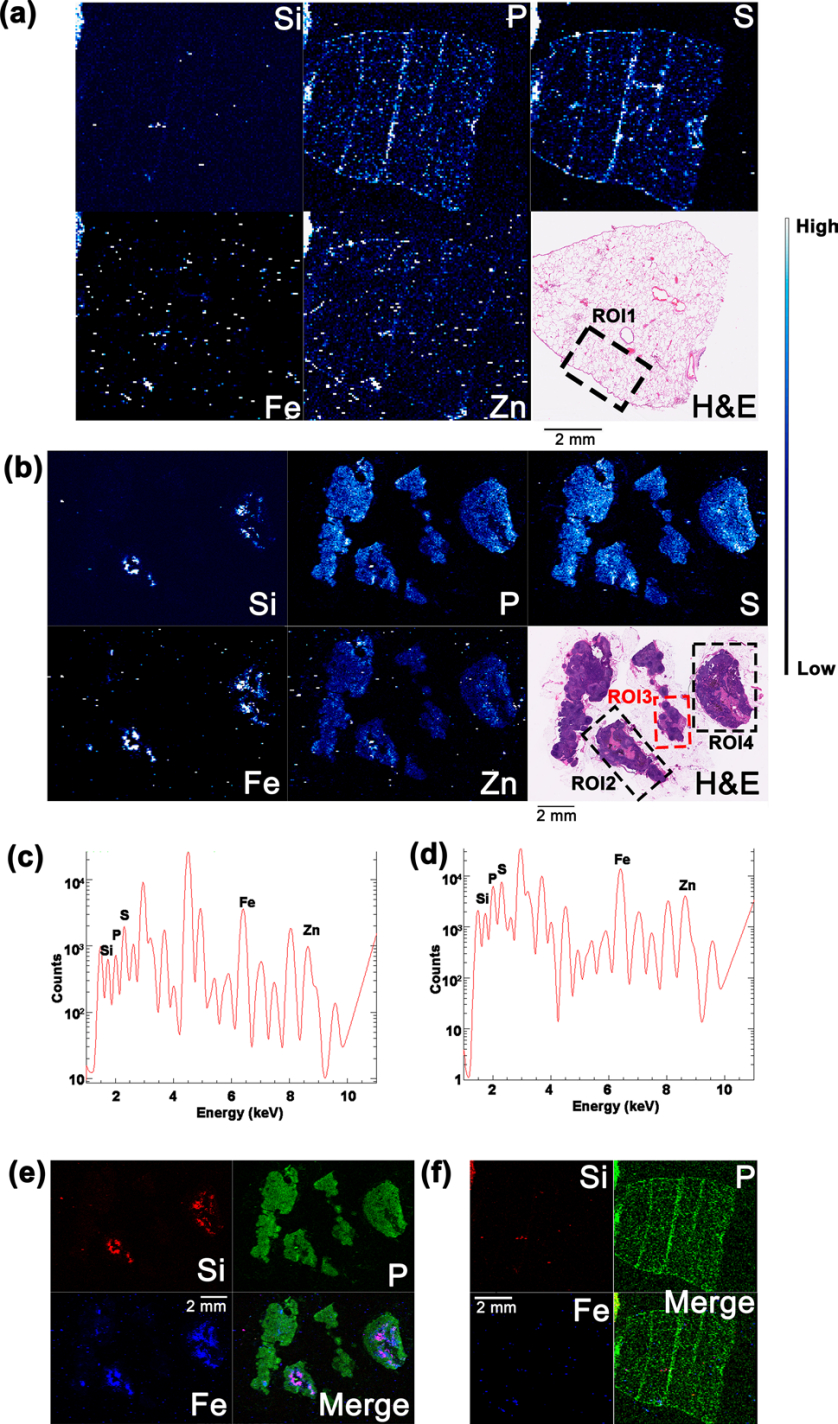
Low-resolution, XFM elemental maps of lung and lymph node tissues from dog 348C (30 μm X-ray beam spot at beamline 8-BM-B). Combined 2D elemental maps and H&E images of lung (**a**) and lymph node (**b**) sections show co-registration of Si, P, S, Fe, and Zn and structure of each tissue. Elemental maps are 15,851 (**a**) and 34,662 (**b**) pixels. K_α_ spectra of X-rays emitted by samples semi-quantitatively confirm the presence of each element in the lung (**c**) and lymph node (**d**). Merged elemental maps show colocalization of Si and Fe in each tissue while P serves as a marker for the entire tissue (**e**, **f**). Black boxes show subsections of sample referred to as *regions of interest* (*ROI*) in each tissue analyzed subsequently with medium- (Figs. 2, 3 & 5) and high-resolution (Fig. 4) XFM. Red box in (**b**) indicates *ROI3* with metastatic spread of bronchoalveolar carcinoma; the specific FFPE tissue block of the lung used in this analysis was cancer free. Color bars next to (**a**) and (**b**) shows that per pixel signal intensity goes from low (black) to medium (blue) to high (white). Scale bars in (**a**), (**b**), (**e**), and (**f**) are 2 mm.

A 30 μm X-ray beam spot generated at beamline 8-BM-B (Table 1), was used with 30 μm steps to generate low resolution elemental maps of archival FFPE specimens that would inform us about the lung and lymph node distribution of ^90^Y-aluminosilicate microparticles that the animal inhaled in 1968. The spatial distribution and relative quantities of Si (from the microparticles) and P, S, Fe, and Zn from the biological material were detected in sections of both tissues with the 8-BM-B beamline (Fig. 1). It should be noted that microparticles also contained Al, but scan energies used at sector 8, as well as higher resolution scans, were not optimized for imaging of Al. At the resolution used, areas with the highest concentrations of Si were observed to co-localize with areas high in Fe. To align different areas of the sample with features identified by pathology evaluation, the elemental map of S was compared to the histological images of each tissue. A co-registration between the omnipresent sulfur signal in the elemental maps which can be used as a proxy for proteins and ubiquitous eosin-staining in the optical images was observed here as before, both in frozen and FFPE specimens^19,22,36^. Additionally, this and subsequent figures show elemental spectra for each sample with element specific peak energies of X-rays emitted from atoms within each sample where peak intensities correspond with signal intensity (Fig. S1b).

**Table 1:** Beamlines and X-ray energies used at the Advanced Photon Source for elemental mapping.

Beamline	Resolution	Beam energy (keV) used	X-ray beam spot size
8-BM-B	Low	11.2	30 μm
2-ID-E	Medium	10.5	0.5 μm
2-ID-D	Medium	17.0	0.3 μm
9-ID-B (BNP)	High	13.7	80 nm

**Figure 2:**
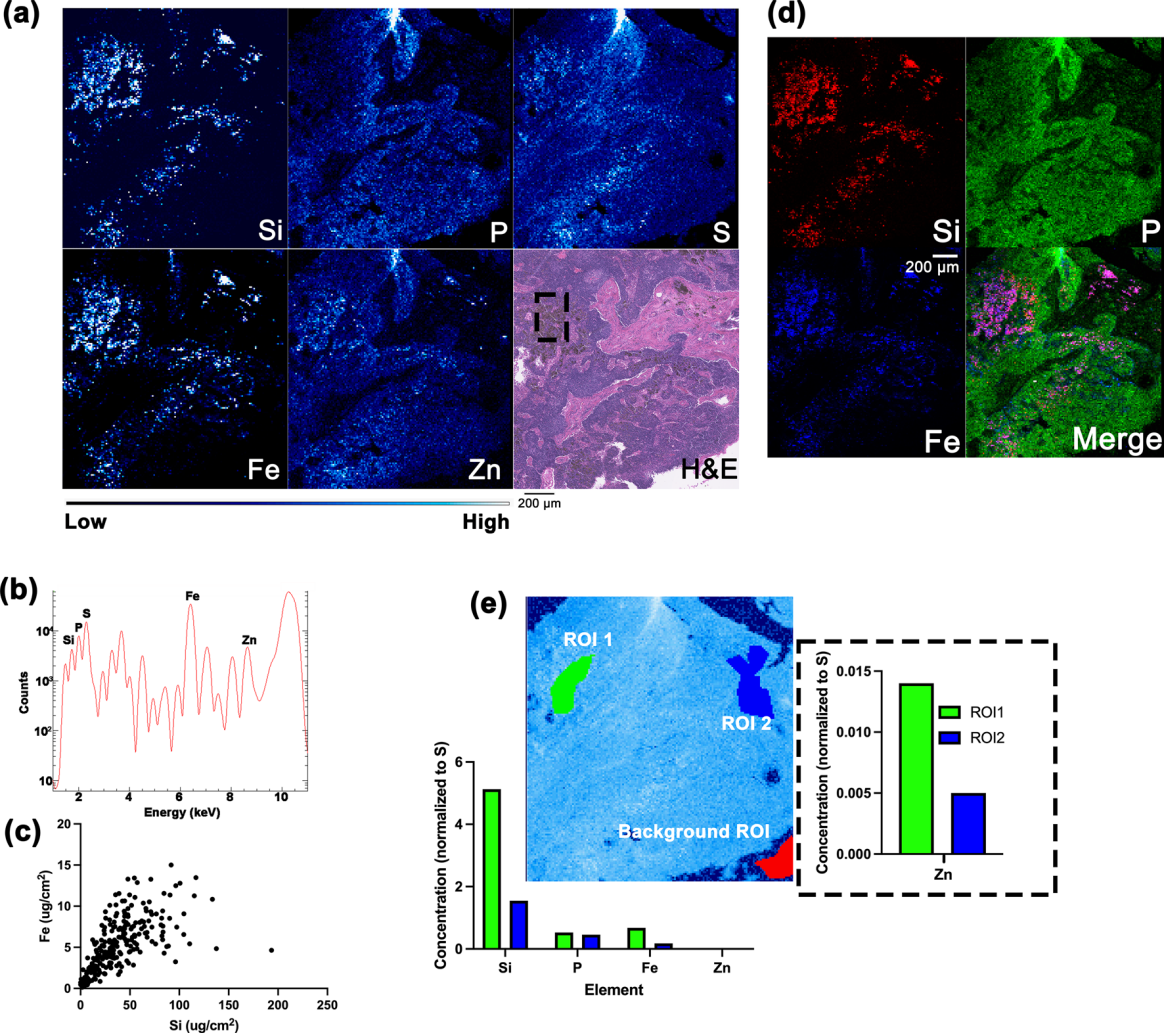
Medium-resolution, XFM analysis of a Si-enriched subsection of *ROI4* (defined in Fig. 1b) from the lymph node (500 nm X-ray beam spot at beamline 2-ID-E). Elemental maps show the distribution of Si, P, S, Fe, and Zn (**a**) with the corresponding H&E-stained image of the lymph node. The elemental maps of the lymph node are 24,311 pixels. Spectrum showing K_α_ lines of different elements present in the sample (**b**). Black box in image (**a**) shows area in H&E-stained lung section containing melanophages that coincides with high elemental signals for Si and Fe; a positive correlation between Si and Fe signals (**c**) in this area was found (Spearman’s rank correlation coefficient ρ is 0.841). Visual representation of colocalization of Si, P and Fe signals is shown in (**d**). Elemental ROI analysis comparing the relative quantity of P, Fe, and Zn between a Si-rich (ROI1)) and a Si-poor area (ROI2). An area without cells (Background ROI) was used to normalize signal calculation. P, Fe, and Zn concentrations are elevated in the Si-rich area relative to the Si-poor area. (**e**). Color bar in (**a**) shows per pixel signal intensity going from low (black) to medium (blue) to high (white). Scale bar in (**a**) is 200 μm.

**Figure 3:**
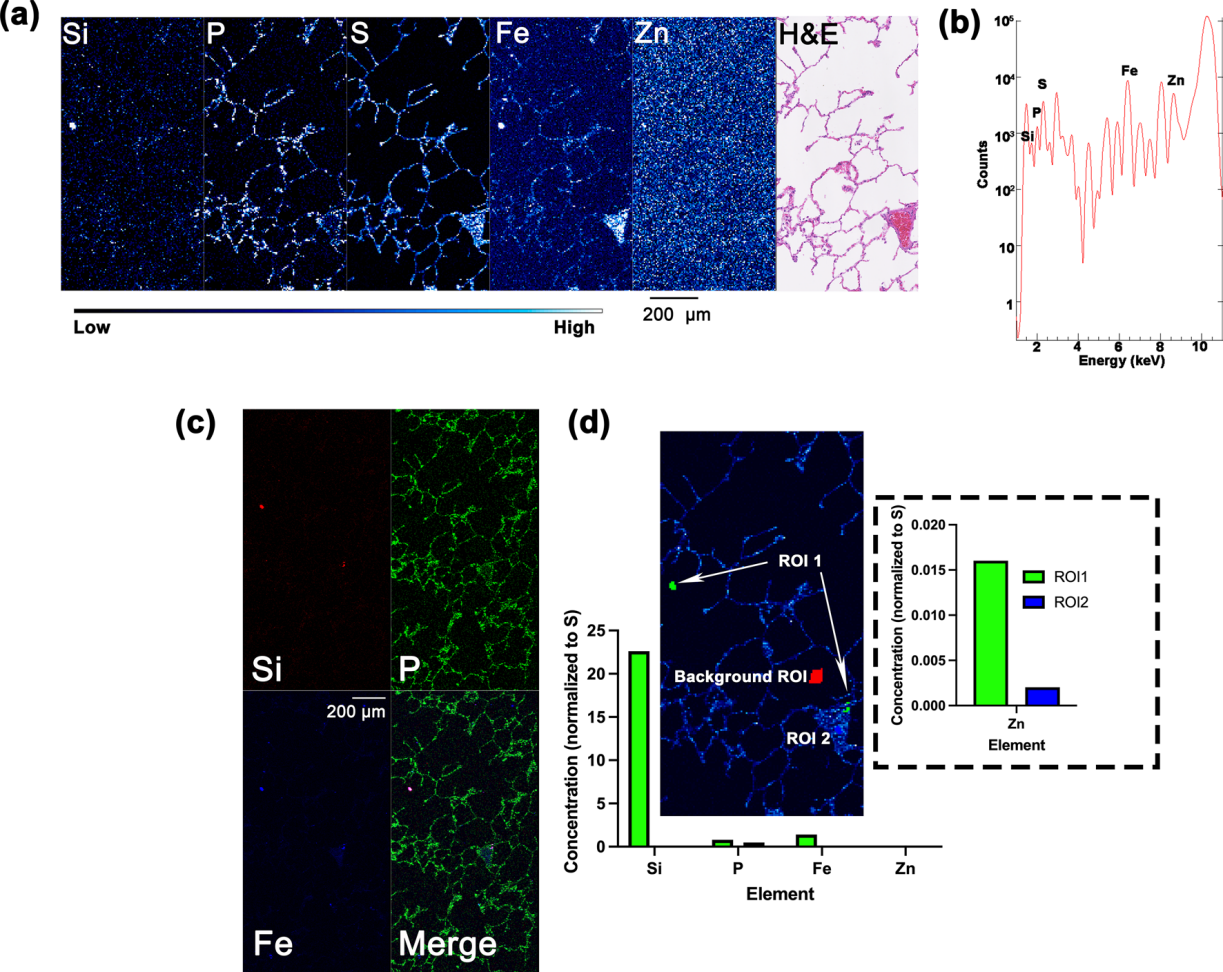
Medium-resolution XFM elemental maps of subsection of *ROI1* (defined in Fig. 1a) from the lung imaged with 500 nm X-ray beam spot at beamline 2-ID-E. The distribution of Si, P, S, Fe, and Zn in 41,601-pixel elemental maps of the lung (**a**); spectrum showing X-ray emission of the sample with positions of K_α_ lines for different elements as indicated (**b**). Three color representation of colocalization between Si, P and Fe signals (**c**). Elemental ROI analysis comparing the relative quantity of P, Fe, and Zn between Si-rich (ROI1) and Si-poor areas (ROI2). An area without cells (BackgroundROI) was used to normalize signal calculation. The area enriched with Si has higher concentrations of P, Fe, and Zn compared to the area containing low Si. (**d**). Color bar in (**a**) shows per pixel signal intensity going from low (black) to medium (blue) to high (white). Scale bar in (**a**) is 200 μm.

To further examine details of the FFPE specimens, samples were reduced in size, remounted and medium resolution XFM was utilized. Based on the low-resolution elemental maps, subsections of the lung and lymph node enriched with Si were selected for further analysis at beamline 2-ID-E (Table 1). Coarse scanning was done using a 0.5 μm beam spot size to raster scan the samples with 10 μm steps. This approach allowed us to save scan time but still obtain sufficient elemental information to generate sample overview images. Overall distribution of Si, S, P, Fe, and Zn in the transbronchial lymph node (Fig. S2) allowed us to align elemental maps with the H&E image. Subsequent medium-resolution elemental mapping with a one-micron step size (Fig. 2, Fig. S3) confirmed results observed in the low-resolution elemental maps (Fig. 1) showing much greater detail of tissue structure and detailed distribution of Si, S, P, Fe, and Zn in the lymph node. Compared to low-resolution XFM, medium resolution XFM allows for improved association between the 2D elemental map and morphological features present in histological images. Interestingly, brown colored, melanin-rich melanophages co-registered with Si in multiple areas of the lymph node (Fig. 2a, S2a, S3a, S3f., S3k, and S3p). At this map resolution, Si-enriched areas co-localize with areas high in Fe in the lymph node (Fig. 2d, S2d, S3d, S3i, S3n, and S3s) and lung (Fig. 3c, S4c, S4f., S4i). Elemental map regions of interest (ROI) analyses in both tissues confirm that representative Si-poor regions contain less Fe compared to Si-enriched regions (Figs. 2e, 3d, S2e, S3e, S3j, S3o, S3t). Spearman’s rank correlation analyses resulted in a positive correlation between Si and Fe signals in areas enriched with melanophages with Spearman’s rank correlation coefficient ρ ranging from 0.663–0.903 (Table 2, Fig. S2c, S2c, S3c, S3h, S3m, S3r).

**Table 2:** Two-tailed Spearman’s rank correlation analysis calculating relationship between Si and Fe signals in melanophage-enriched areas of the lymph node (*α* = 0.05).

Figure	Spearman’s rank correlation coefficient ρ	95% confidence interval	*P* value	# XY pairs
Fig. 2c	0.841	0.802 to 0.872	< 0.0001	290
Supplementary Fig. S2c	0.903	0.827 to 0.947	< 0.0001	45
Supplementary Fig. S3c	0.885	0.868 to 0.899	< 0.0001	842
Supplementary Fig. S3 h	0.663	0.646 to 0.679	< 0.0001	4629
Supplementary Fig. S3 m	0.873	0.866 to 0.880	< 0.0001	4732
Supplementary Fig. S3r	0.834	0.830 to 0.838	< 0.0001	23,738

A lung FFPE sample was also scanned at the medium resolution instrument with a step size of 10 μm, which was greater than the focused beam spot size (0.5 μm) used to generate overview maps that guided selection of areas of interest for detailed scanning (Fig. 3, Fig. S4) using the H&E images as a reference. Some tissue features not noticeable in the much more cellular lymph node sample were very apparent in the lung. For example, the eosin-positive, hematoxylin-negative areas of the lung that correspond with red blood cells co-registered with high Fe and S signals (Fig. 3a, S4a, S4d) as previously observed^37,38^. A few Si rich regions of small size were noticed in lung samples, likely corresponding to small aggregates or even single microparticles. These co-localized with high Fe signal as well (Fig. 3, Fig. S4).

**Figure 4:**
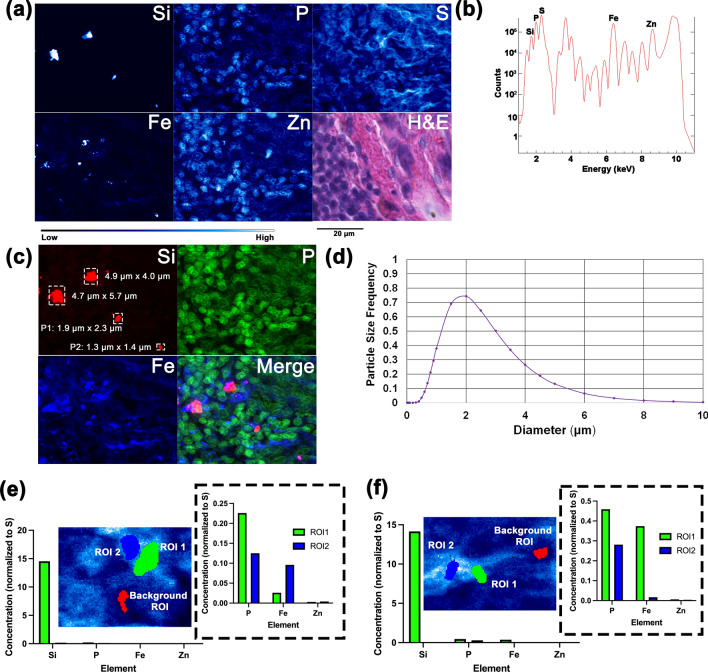
BNP was used to identify Si-containing microparticles in the lymph node from *ROI2* (defined in Fig. 1b) using high-resolution XFM (80 nm X-ray beam spot size). Elemental mapping (**a**) and spectrum of K_α_ X-rays produced by the sample (**b**) detects Si, P, S, Fe, and Zn in 289,081-pixel images. Three color colocalization of Si, P and Fe signals (**c**) displays Si signals that may correspond to potential aggregates of aluminosilicate particles and single aluminosilicate particles (P1 and P2). Particle size distribution analysis of aluminosilicate particles generated at ITRI (**d**) are based on the publication by Rabbe and others^39^. Approximate diameters of P1 and P2 measured using MAPS software (**c**) fall within the particle size distribution of aluminosilicate particles. Elemental ROI analysis of P1 and P2 shows the relative elemental concentrations of Si, P, Fe, and Zn in Si-rich (ROI1) and neighboring Si-poor (ROI2) regions of similar size (Methods). Small regions with overall low signals serve as background for subtraction (Background ROI). Si-rich regions no longer correlate with P and Fe in the same way as they did at medium resolution. Color bar in (**a**) shows per pixel signal intensity going from low (black) to medium (blue) to high (white). Scale bar in (**a**) is 20 μm.

Subcellular-level elemental mapping of the lymph node was completed using the BNP at beamline 9-ID-B (Table 1). High-resolution elemental maps of Si, P, S, Fe, and Zn provided much more detail than the low- and medium-resolution XFM data (Fig. 4a, c). For example, the discrete five-micron diameter features that matched hematoxylin staining of cell nuclei, had a more punctate P signal as previously shown^21^. Thus, while nuclei were clearly noticeable in medium resolution images obtained with one-μm step size, in BNP images, correspondence of P signal with chromatin became even more pronounced. Interestingly, high resolution map registered several distinct Si rich areas of 1.5–10 μm in size that could correspond to single microparticles, or, in the case of 10 μm structures, small aggregates or single particles. Importantly, at this resolution it became clear that the Fe signal is often only adjacent to, but not fully overlapping with the Si signal (Fig. 4c, e). The approximate size of two of these Si areas are 1.9 × 2.3 μm^2^ and 1.3 × 1.4 μm^2^ (Fig. 4c), matching the sizes of single aluminosilicate particles.

**Figure 5:**
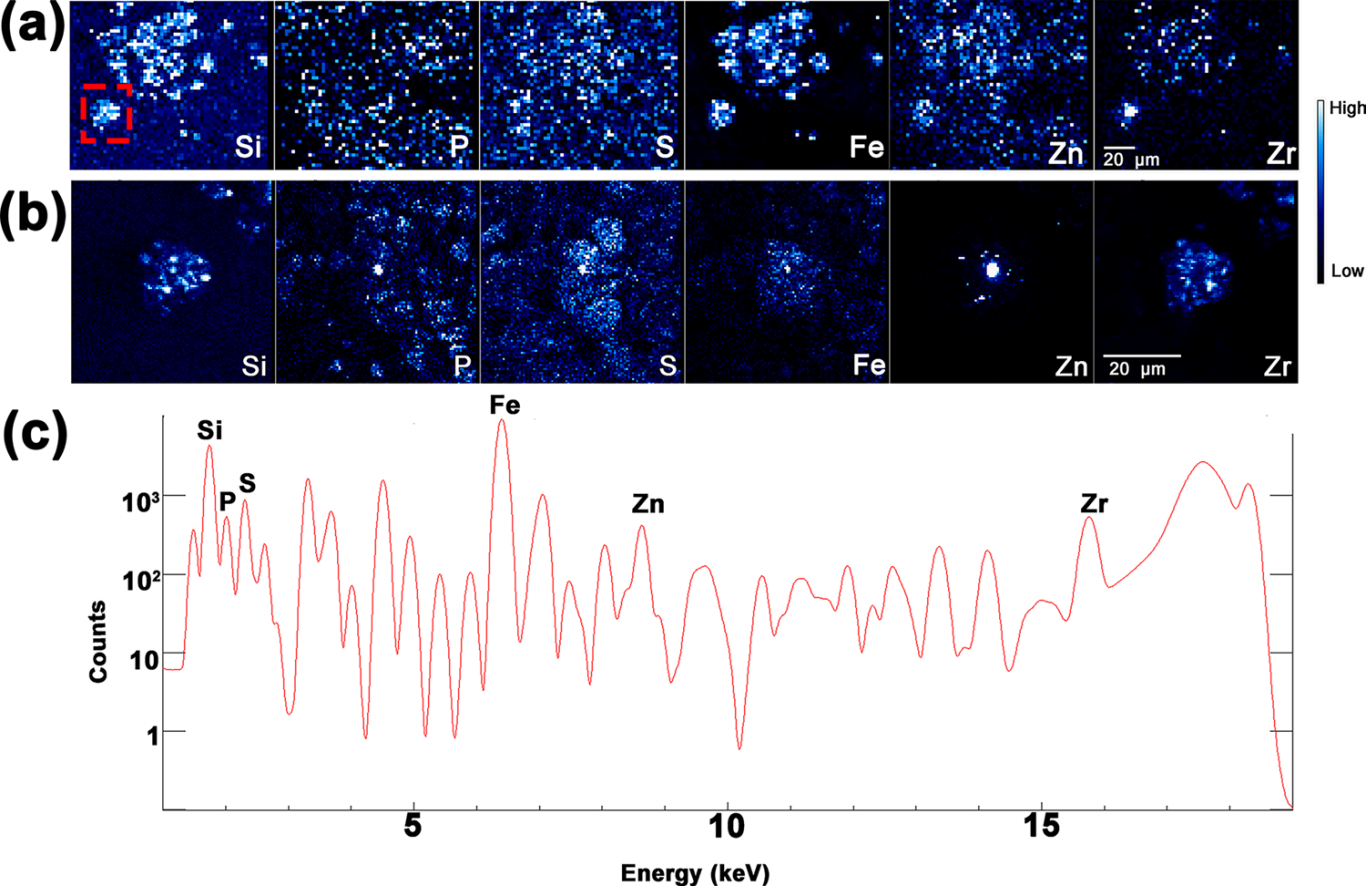
A lymph node sample was scanned with X-rays tuned to 18.3 keV energy at the 2-ID-D beamline (X-ray beam spot size of 300 nm). Overview of a larger sample area (**a**) and a small sub-area of the sample (**b**) show elemental distribution of Si, P, S, Fe, Zn, and Zr. Red box in (**a**) indicates area examined in (**b**). Images are 3111 (**a**) and 5100 pixels (b). Complete spectrum displays K_α_ elemental signature of (**a**) and counts of each element detected. Scale bars in (**a**) and (**b**) are 20 μm.

Considering the number of microparticles per area of tissue in the lymph node was far greater than in the lung, and that phagocytosing cells transfer the microparticles to the lymph nodes in low pH vacuoles, it was of interest to determine whether the microparticles still retained their elemental composition during transport. Because ^90^Y in the FAPs has decayed to ^90^Zr, we subjected the lymph node sample to X-ray imaging at 18.3 keV to excite K_α_ fluorescence of Zr. X-ray energies used for scans presented in Figs. 1, 2, 3 and 4 were below this value and could not excite fluorescence of the K_α_ shell of Zr. Overview and detailed lymph node elemental maps obtained at a medium resolution station 2-ID-D (Table 1) and the X-ray spectrum (Fig. 5) showed the presence of Zr in the sample, in the regions overlapping with Si signal. This finding suggests that the microparticles remained stable inside cells and that the transit of “insoluble radionuclides” was indeed dependent on transport of microparticles through the body.

## Discussion

The FFPE specimens from a lifespan study designed to follow the effects of inhalation exposure to insoluble radionuclides conducted at ITRI in 1968 were analyzed in detail using synchrotron-based XFM. Overall, this study included 89 dogs that were exposed to ^90^Y adsorbed to FAPs^6–9^. This radionuclide has a short half-life of 2.6 days. Due to delivery by inhalation, and phagocytosing cells taking hours or even days to travel to draining lymph nodes^43^ most of the dose was delivered to the lung. The ^90^Y lifespan study estimated the initial lung burden in different animals to be between 2.96 and 192 MBq/kg of body weight, with cumulative doses to the lung ranging from 13–700 Gy. Acute lung injury was the cause of death in 40 of the animals that received cumulative lung doses between 48 and 700 Gy and died 7.5 to 4559 days post inhalation. Of the remaining 49 beagles, 10 died between 2250–6084 days after exposure from lung neoplasia. These dogs received cumulative lung doses between 19 and 100 Gy. The dog selected for this study, dog ID 348C, falls in the latter category—the cumulative lung dose it received was 57 Gy and the animal died from bronchoalveolar carcinoma of the lung 5366 days after inhalation. At the time of exposure this male beagle was 376 days old, weighed 8.7 kg and the dose of ^90^Y it inhaled with fused aluminosilicate particles produced an initial body burden of 670 μCi/kg.

We used low-, medium- and high-resolution (Table 1) synchrotron-based XFM to produce elemental maps of the tissues and cells, as well as 2D images of single FAP microparticles. The spatial distribution of P, S, Fe, Zn, and Si (Figs. 1, 2, 3 and 4) is shown in the lung and lymph node tissues. The distribution of Zr in lymph node is also shown (Fig. 5). Sulfur-containing amino acids are present in all cellular proteins and P is an essential structural element of nucleic acids. Consequently, S and P maps highlight the tissue overall and cell nuclei of each specimen, respectively (Figs. 1, 2, 3, 4 and 5, S2–S4). Both lung and lymph node tissues contain areas rich in Fe and Si. To determine if Si signals are representative of FAPs, high resolution XFM was performed. The Si-enriched regions of 1.9 × 2.3 μm^2^ and 1.3 × 1.4 μm^2^ were detected and are within the reported size of fused aluminosilicate particles produced by ITRI researchers^39^. There is no endogenous Si in the body; therefore, the Si-containing microparticles detected are the remnant of insoluble FAPs carrying radioactive ^90^Y. We believe this to be the first example of XFM use to detect individual aluminosilicate particles in an archival canine tissue sample.

While ^90^Y decays quickly, and most of the dose was deposited in the lung tissue, XFM images show that the density of aluminosilicate particles in the lung is low, with most of the particles re-distributed to  tracheobronchial lymph nodes over time. These results suggest that insoluble radionuclides inhaled during radiological emergencies can be expected to remain inside the body for extended periods of time; for those radionuclides with long half-life such as for example plutonium, particles would continue to be a source of ionizing radiation exposure for the duration of animals’ life. The average diameter of radioactive microparticles from the Chernobyl nuclear accident was 2–10 µm which may have been inhaled by radiation workers, first-responders, and members of the public^40^. The size of those microparticles is similar to the particle range that was used for experiments done at ITRI.

At low and medium resolution XFM we observed co-localization of Fe and Si signals in the lung and lymph node specimens, resolving into adjacent Si and Fe signals when imaging was done at the highest resolution. The microparticles are likely re-distributed from lung to lymph nodes by phagocytosing cells. Considering that there is a measurable quantity of Fe in macrophages^41^ and that the macrophages are both radioresistant and active in phagocytosis of foreign substances including microparticles^42^ it is likely that the colocalization of Si and Fe is a result of microparticle uptake by macrophages. In H&E images of  tracheobronchial lymph nodes pathologists reported the presence of melanophages in the areas of sample corresponding with the highest Si and Fe signals.

There is an unmet need to identify novel biomarkers of radiation exposure to refine the medical response to mass casualty radiation events. NURA contains FFPE specimens from numerous radiobiology experiments making it an invaluable resource for ancillary, biomarker discovery studies. Current biomarkers of radiation exposure are based on studies involving external beam radiation; to date, the scientific community has not identified biomarkers of internal radiation exposure. The data from this proof-of-principle study showcases XFM as a reliable technique for investigation of decades-old archival samples enabling future studies exploring markers of internal radiation exposure using NURA samples.

## Methods

### Specimens

Lung and  tracheobronchial lymph node FFPE tissues from a single beagle (NURA Dog ID 348C) were retrieved from the Northwestern University Radiobiology Archive. For an overview about the approaches used to work with the animals, consult^6–9^. Portions of lung tissue and lymph node from the location adjacent to the division of trachea and main bronchi were harvested from a moribund dog that experienced a single inhalation of ^90^Y adsorbed to fused aluminosilicate particles (FAPs) (Fig. S1a). After processing in Bouin’s solution, the tissues were dehydrated, and paraffin embedded as described^6–9^. Two serial, immediately adjacent seven-micron thick sections were generated from each FFPE block using a Leica microtome. One pair of lymph node tissue sections and one pair of lung tissue sections were generated and used for this work. An additional, single section of lymph node tissue was examined for the presence of Zr. One section was placed on a glass slide and stained using hematoxylin and eosin (H&E) while the immediately successive tissue section was placed on sample support (Ultralene membrane, SPEX, supported by an in-house generated 3D printed frame) that is optimal for X-ray fluorescence microscopy analysis as detailed in the next section. Additional glass slide for H&E staining for co-registration of histological images and XFM analysis of Zr was not prepared. Images of the H&E-stained tissues were reviewed by StageBio, independent pathology contractor company. According to their evaluation—metastatic spread of bronchoalveolar carcinoma was detected in transbronchial lymph node, while the specific lung tissue FFPE sample used for this study did not contain cancer foci.

Detailed information about the production of aluminosilicate particles was provided in Raabe and others^39^. In short, the particles were generated from montmorillonite (Si_8_Al_4_O_20_(OH)_4_ + nH_2_O) at high temperature which led to generation of microparticles between 0.5 and eight μm in size; particles with 1.8 μm diameter were the most frequent. Each gram of microparticles contained 2578 µg of ^90^Y; this corresponded to a 100-fold difference between Y and Si and an expected 10 femtograms of Y and one picogram of Si in a particle of average size. Considering that _39_Y^90^ decays to _40_Zr^90^ with a half-life of 2.669 days while radioactive inhalation exposure occurred in 1968, the same microparticles today contain Zr to Si ratio of 1:100.

### Low resolution elemental mapping

Custom sample holders were created using a 3D printer, permitting sample repositioning to allow for a most efficient XFM imaging at the 8-BM-B beamline at the APS at ANL. Tissue sections were adhered to the Ultralene membranes (SPEX Sample Prep, LLC, 15 Liberty St., Metuchen, NJ 08,840, USA) assembled within each holder. Low-resolution elemental mapping of each specimen was completed using a 30 μm beam spot. Each specimen was raster-scanned using 11.2 keV hard X-rays with dwell times of 50 ms per pixel. A SII Vortex ME4 4-element silicon drift detector (SII NanoTechnology USA, Northridge, CA) was utilized to collect spectra. Calibration of elemental concentrations was carried out using AXO 10X thin film AXO standards (Applied X-ray Optics (AXO), Dresden, GmbH, Germany)^35^. Data analysis was completed using MAPS software.

### Medium resolution mapping

Low resolution elemental maps acquired at the 8-BM-B beamline at APS were analyzed and elemental maps were used to select areas of each tissue with heterogeneous distribution of Si as a proxy for aluminosilicate particles. Selected sample regions were excised and placed onto sample holders suitable for medium resolution imaging at the 2-ID-E and 2-ID-D beamlines, where imaging was done with a 0.5 μm beam spot size at energy of 10.5 keV and with 0.3 μm beam spot size at energy of 18.3 keV, respectively. Fresnel zone plate optics (X-radia, Concord, CA) were used to focus the X-rays. Areas of each sample were raster scanned using 50 ms (2-ID-E) or 100 ms (2-ID-D) dwell times to collect fluorescence spectra for each pixel (Fig. S1b). Fluorescence signals were calibrated to per pixel elemental concentrations using thin-film standards NBS-1832 and NBS-1833 (National Bureau of Standards, Gaithersburg, MD), AXO 1X (AXO Dresden, GmbH, Germany), and MAPS software^35^. Final analysis was completed using MAPS software.

### High resolution mapping

Low resolution XFM data was used to identify a region of the lymph node specimen with high Si content, and the portion of the sample was excised and mounted in sample holder suitable for imaging using the BNP instrument at beamline 9-ID-B at APS. Stacked Fresnel zone plates were used to focus monochromatic X-rays of 13.7 keV to an ~ 80 nm spot. The per pixel dwell time was 50 ms. Similar to the low- and medium-resolution mapping, full spectra were collected from each scanned pixel with an energy dispersive detector and analyzed with MAPS software^35^.

### Elemental ROI analysis & correlation analysis

Regions of interest (ROI) for elemental analyses were selected in the following way: areas without any elemental signals were selected as background ROIs, Si-enriched areas were ROIs containing numerous Si high pixels, and Si-poor area ROIs were areas without notable Si content. For each element, the mean per pixel intensity of each element of interest in background ROI was subtracted from the mean per pixel intensity of each element in test ROIs. The relative concentration of each element was calculated by normalizing the concentration of each element against sulfur to account for varied cell density in each ROI^22^. ROI analyses were done using MAPS software^35^.

Two-sided, Spearman’s rank correlation analysis was used to calculate the significance of correlation between Si and Fe signals in areas of the lymph node containing melanophages. GraphPad Prism 9 software was used to calculate Spearman’s rank correlation coefficients ρ, 95% confidence intervals, p values and generate scatterplots.

## Supplementary Information

Supplementary Information.

## Data Availability

The data sets supporting the findings of the current study are available from the corresponding author upon reasonable request.
