# Supplementary material for: Proof of principle study: synchrotron X-ray fluorescence microscopy for identification of previously radioactive microparticles and elemental mapping of FFPE tissues
Source: Sci Rep. 2023 May 13;13:7806. doi: 10.1038/s41598-023-34890-6 (PMC10183016; doi:10.1038/s41598-023-34890-6)
Supplement: Supplementary file 1 — Supplementary Information. [file 41598_2023_34890_MOESM1_ESM.pdf]

Supplementary data:

Proof of principle study: synchrotron X-ray fluorescence microscopy for identification of previously radioactive microparticles and elemental mapping of FFPE tissues

Letonia Copeland-Hardin[1], Tatjana Paunesku[1], Jeffrey S. Murley[1], Jasson Crentsil[1], Olga Antipova[2], LuXi Li[2], Evan Maxey[2], Qiaoling Jin [2], David Hooper[3], Barry Lai[2], Si Chen[2], [*]Gayle E. Woloschak[1]

[1]Department of Radiation Oncology, Feinberg School of Medicine, Northwestern University, Chicago, IL, United States
[2]X-ray Science Division, Advanced Photon Source, Argonne National Laboratory, Lemont, IL, United States
[3]Nuclear Nonproliferation Division, Oak Ridge National Laboratory, Oak Ridge, TN, United States
[*]corresponding author

Supplementary Figure 1

(a)

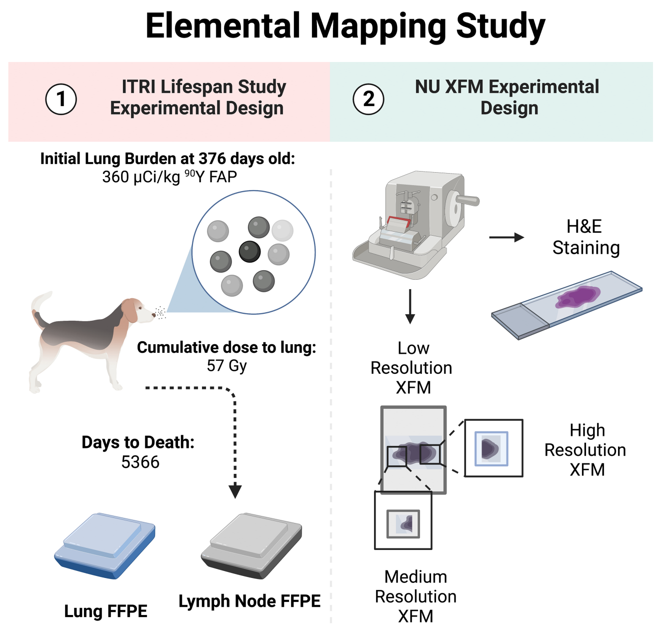

(b)

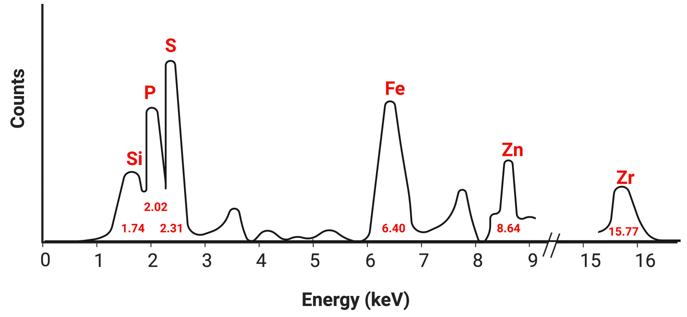

Schematic diagrams illustrating the experimental design used for the Lifespan Beagle Study at the Inhalation Toxicology Research Institute (ITRI) and the current X-ray fluorescence microscopy (XFM) study at Northwestern University (a) and example XFM spectrum (b). Dog 348C inhaled ^{90}Y fused aluminosilicate particles (FAPs) at the age of 376 days. Following necropsy at 5366 days after inhalation, portions of lung and lymph nodes were preserved as formalin-fixed, paraffin-embedded (FFPE) specimens. Currently, these samples are stored in the Northwestern University Radiobiology Archive (NURA). These FFPE specimens were used for the elemental mapping study. Consecutive sections of lung and lymph node FFPE specimens were produced by microtome sectioning and placed on glass slides for H&E staining or on Ultralene membrane for X-ray fluorescence imaging. X-ray emission spectrum displaying elements identified in lung and lymph node samples and corresponding K_α lines (b). Supplemental figures 1(a) and 1(b) were created with BioRender.com.

Supplementary Figure 2

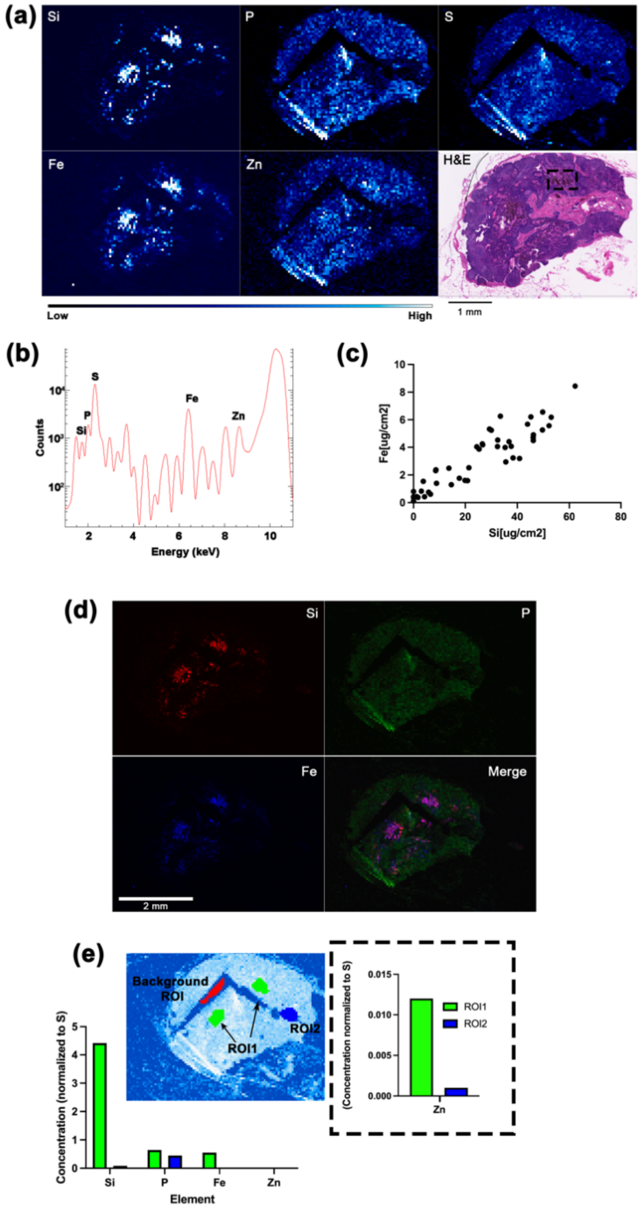

Combined XFM elemental maps and H&E images of formalin-fixed, paraffin-embedded transbronchial lymph node section from dog 348C obtained at 2-ID-E with 500 nm X-ray beam spot size. Overview 2D elemental map of the distribution of Si, P, S, Fe, and Zn in the lymph node was obtained using 500 nm X-ray beam spot in combination with larger scan step size (a). The elemental maps are 24,311 pixels. X-ray spectrum generated by the sample X-ray emission with K_α lines labeled to identify individual elements (b). Black box in (a) shows an area containing melanophages; ROI analysis of this region shows a positive correlation between Si and Fe signals (c) with the calculated Spearman's rank correlation coefficient ρ of 0.903. Three color colocalization image of Si, P and Fe signals (d). ROI analysis was done using a sample free area (red ROI) as background and comparing a Si-rich (green ROI) and a Si-poor (blue ROI) area with respect to the relative quantity of P, Fe, and Zn (e). Relative concentrations of P, Fe, and Zn are elevated in the Si-enriched area compared to the Si-poor area. Color bar in (a) shows the per pixel signal intensity going from low (black) to medium (blue) to high (white). Scale bar in (a) is 1 mm, in (d) 2 mm.

Supplementary Figure 3

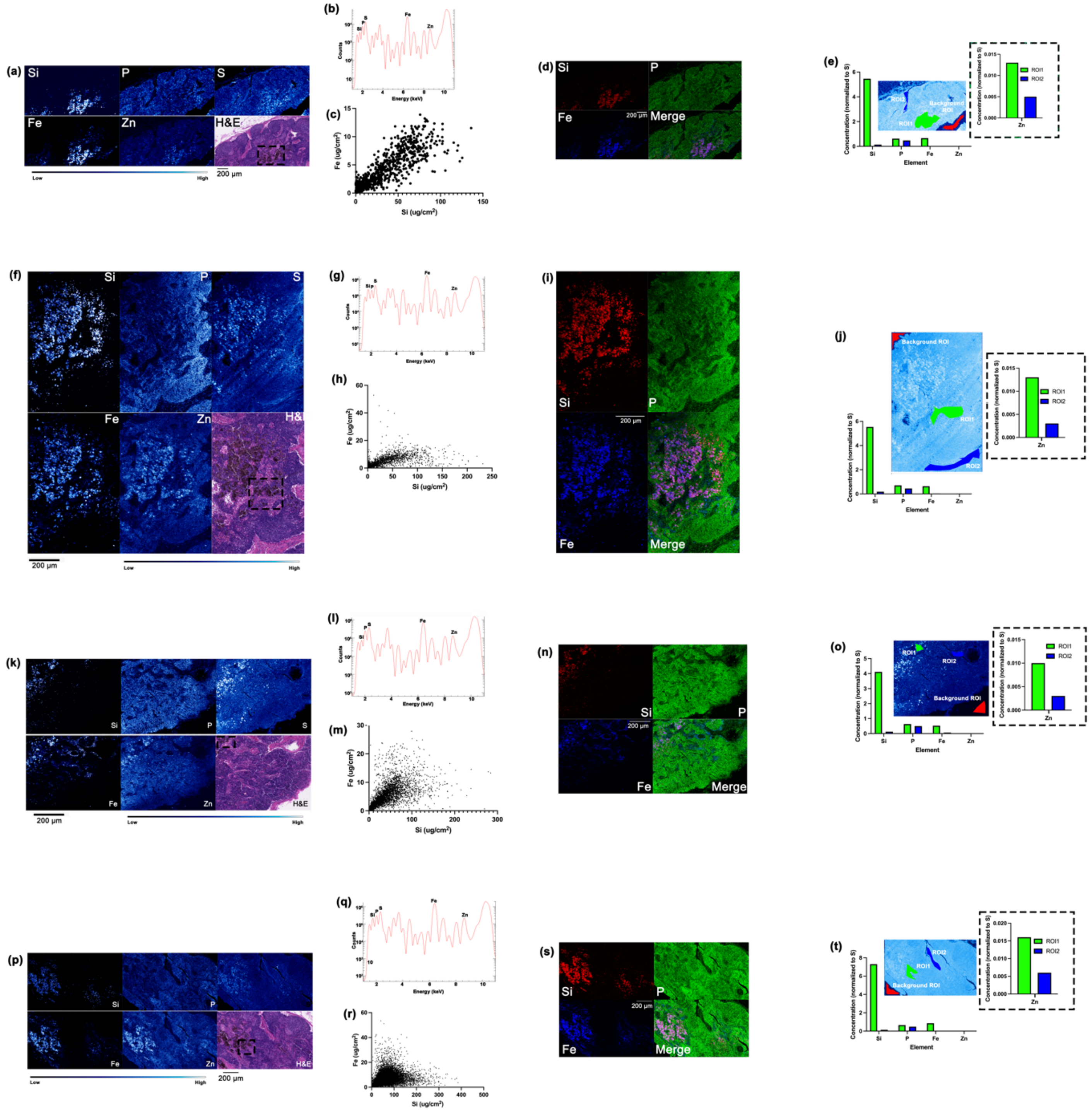

Combined XFM elemental maps and H&E images of FFPE transbronchial lymph node section from dog 348C obtained at 2-ID-E with 500nm beam spot size and 1 µm step size. Medium resolution 2D elemental maps show the distribution of Si, P, S, Fe, and Zn in the lymph node (a, f, k, & p). X-ray spectra showing K_{α} lines indicating elemental composition (b, g, l, & q). Black box (a, f, k, & p) shows area in H&E-stained lymph node section containing melanophages and a positive correlation between Si and Fe signals (c, h, m, & r) in this area, Spearman's rank correlation coefficients ρ are 0.885, 0.663, 0.873, and 0.834 respectively. Three color colocalization maps for Si, P and Fe signals visually show signal colocalization for Si and Fe (d, I, n, & s). ROI analyses comparing Si-rich (ROI1) and a Si-poor areas (ROI2), with sample-free areas as background (Background ROI) for the relative quantity of P, Fe, and Zn (e, j, o, & t). Si-enriched areas contain higher P, Fe, and Zn than Si-poor areas. Color bars in (a, f, k, & p) show per pixel signal intensity going from low (black) to medium (blue) to high (white). The elemental maps are 16,544 (a), 898,464 (f), 940,896 (k), and 1,445,121 (p) pixels. Scale bars in (a, f, k, & p) are 200 µm.

Supplementary Figure 4

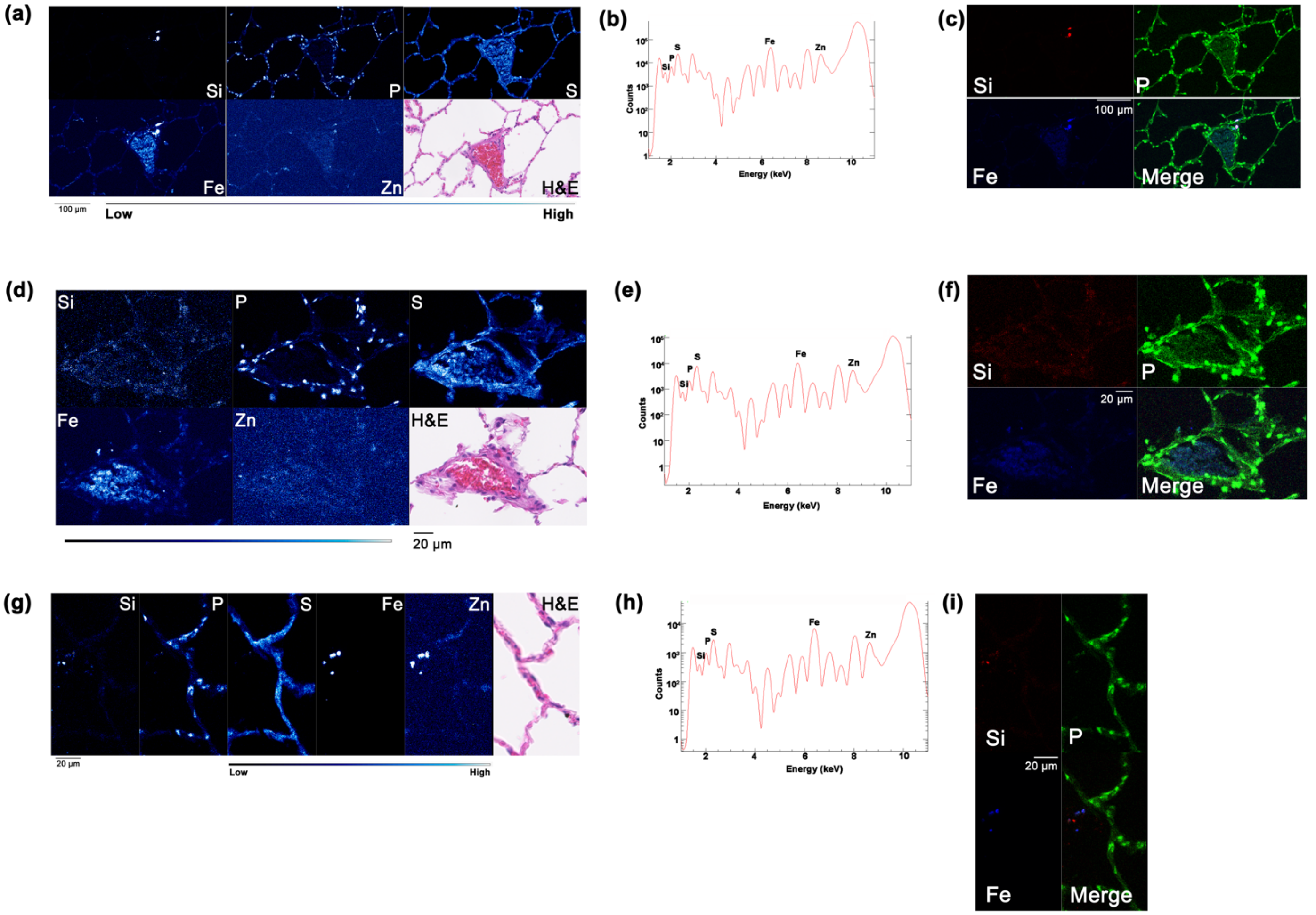

Combined XFM elemental maps and H&E images of FFPE lung sections from dog 348C obtained at 2-ID-E. Medium resolution, 2D elemental maps show the distribution of Si, P, S, Fe, and Zn in the lung (a, d, & g). Images of elemental maps are 530,721 (a), 87,001 (d), and 36,801 (g) pixels. X-ray spectra generated by excitation of the sample with X-ray beam are labeled with positions of K_α lines for different elements present in the sample (b, e, & f). Three color colocalization of Si, P and Fe signals (c, f, & i). Color bars in (a, d & g) show per pixel signal intensity going from low (black) to medium (blue) to high (white). Scale bars in (a), (d) and (g) are 100 µm, 20 µm, and 20 µm, respectively. N.B. ROI analyses were not done because Si rich areas consisted of relatively few pixels.
